# Proteinuria in Deceased Diabetic Donors and Kidney Transplant Outcomes

**DOI:** 10.1177/20543581261424568

**Published:** 2026-02-26

**Authors:** Christie Rampersad, S. Joseph Kim

**Affiliations:** 1 Ajmera Transplant Centre, Toronto General Hospital, University Health Network, ON, Canada; 2 Institute of Health Policy, Management and Evaluation, University of Toronto, ON, Canada

**Keywords:** kidney transplant, diabetes mellitus, proteinuria, graft survival, mortality

## Abstract

**Background:**

Diabetes mellitus is increasingly common among deceased donors and may signal donor-derived kidney injury that affects post-transplant outcomes.

**Objective:**

To evaluate whether donor proteinuria is associated with graft and patient outcomes after kidney transplantation from deceased donors with diabetes.

**Design:**

Retrospective cohort study using a national transplant registry.

**Setting:**

United States; Scientific Registry of Transplant Recipients (SRTR), February 28, 2013–2023.

**Patients:**

9486 kidney-alone transplant recipients from deceased donors with diabetes in whom a pre-implantation (procurement) biopsy was performed and donor proteinuria status was available.

**Measurements:**

Primary outcome: death-censored graft failure (DCGF). Secondary outcomes: all-cause graft failure (ACGF), death with graft function (DWGF), and delayed graft function (DGF). Exposure: donor proteinuria (present vs absent).

**Methods:**

Kaplan-Meier analyses and multivariable Cox models (a priori covariables from a directed acyclic graph) assessed associations between donor proteinuria and time-to-event outcomes. Because proportional hazards were violated for DCGF, analyses were performed in two periods: an “early” cohort up to 2.5 years post-transplant and a landmarked cohort of recipients with functioning grafts at 2.5 years. Logistic regression evaluated DGF. Sensitivity analyses adjusted for donor insulin dependence (proxy for diabetes severity) and recipient characteristics; exploratory effect modification by biopsy glomerulosclerosis (GS) was assessed.

**Results:**

Donor proteinuria was present in 54.9% of cases. In adjusted Cox models, donor proteinuria was not associated with early DCGF (<2.5 years; HR 1.14, 95% CI: 0.99, 1.32) but was associated with increased risk of late DCGF >2.5 years post-transplant (HR 1.36, 95% CI: 1.15, 1.62), with similar findings for ACGF. No associations were observed with DWGF or DGF. Results were consistent after adjustment for donor insulin dependence as a proxy for severity and recipient factors including diabetes status. The association between proteinuria and late graft failure was more pronounced in kidneys with lower GS, suggesting proteinuria may reflect chronic injury not well-captured by biopsy.

**Limitations:**

Observational design with potential residual confounding. Because the cohort includes only kidneys that were actually transplanted, findings reflect outcomes among accepted organs and are not intended to guide offer acceptance or decline decisions. Donor proteinuria was recorded only as present or absent, without standardized measurement. This may have led to misclassification, prevented assessment of dose-response relationships, and likely made it harder to detect true associations. Registry constraints limited histologic detail beyond GS.

**Conclusions:**

Among diabetic deceased donors, the presence of proteinuria is a time-dependent marker of increased long-term graft-failure risk, complementing biopsy and clinical data. Standardized, quantitative proteinuria assessment may improve risk stratification and post-transplant management while supporting judicious utilization of diabetic donor kidneys.

## Abrégé

**Contexte:**

La prévalence croissante du diabète chez les donneurs décédés pourrait être le signe d’une atteinte rénale transmise par le donneur et susceptible de compromettre les résultats de la transplantation.

**Objectifs:**

Évaluer l’association entre la présence d’une protéinurie chez le donneur et les résultats de la greffe et des receveurs après la transplantation de reins provenant de donneurs décédés diabétiques.

**Conception:**

Étude de cohorte rétrospective à partir d’un registre national de transplantation.

**Cadre:**

États-Unis; Scientific Registry of Transplant Receivers (SRTR), 28 février 2013 à 2023.

**Sujets:**

Les 9 486 receveurs d’une greffe rénale seule, dont l’organe provenait d’un donneur décédé diabétique, pour lesquels une biopsie préimplantatoire (au moment du prélèvement) a été réalisée et dont le statut de protéinurie du donneur était disponible.

**Mesures:**

Critère d’évaluation principal: échec de la greffe censurée par le décès (ÉGCD). Résultats secondaires: échec de la greffe toutes causes confondues (ÉGTC), décès avec greffon fonctionnel (DAGF) et reprise retardée de la fonction du greffon (RRFG). Exposition: protéinurie du donneur (présente c. absente).

**Méthodologie:**

Des analyses de Kaplan–Meier et des modèles de Cox à variables multiples (avec covariables a priori issues d’un graphe acyclique orienté) ont servi à évaluer les associations entre la protéinurie du donneur et les événements survenus dans le temps. Puisque l’hypothèse des risques proportionnels n’était pas respectée pour l’ÉGCD, les analyses ont été divisées en deux périodes: une cohorte « précoce » couvrant les 2,5 premières années après la greffe, et une cohorte « Landmark » portant sur les receveurs dont le greffon était toujours fonctionnel à 2,5 ans. La régression logistique a servi à évaluer les DAGF. Les analyses de sensibilité ont tenu compte de l’insulinodépendance du donneur (indicateur de la gravité du diabète) et des caractéristiques du receveur; le possible effet modificateur de la glomérulosclérose (GS), observée à la biopsie, a été exploré.

**Résultats:**

Le donneur présentait une protéinurie dans 54 % des cas. Dans les modèles de Cox corrigés, la protéinurie du donneur n’a pas été associée à un ÉGCD précoce, soit moins de 2,5 ans après la greffe (RR: 1,14; IC 95 %: 0,99-1,32), mais à un risque accru d’ÉGCD tardif, soit plus de 2,5 ans après la greffe (RR: 1,36; IC 95 %: 1,15-1,62), avec des résultats similaires pour l’ÉGTC. Aucune association n’a été observée avec un DAGF ou une RRFG. Ces résultats se sont maintenus après la correction pour l’insulinodépendance du donneur (indicateur de gravité) et les caractéristiques du receveur, y compris le diabète. L’association entre la protéinurie et l’échec tardif de la greffe était plus marquée dans les cas où le rein présentait une faible GS, ce qui suggère que la protéinurie pourrait être le reflet de lésions chroniques que la biopsie ne permet pas de bien évaluer.

**Limites:**

Étude observationnelle pouvant présenter un biais de confusion résiduel. La cohorte s’est limitée aux reins ayant été transplantés; les conclusions portent donc exclusivement sur les organes acceptés, et ne doivent pas servir à orienter les décisions d’acceptation ou de refus d’organes. La protéinurie du donneur a été enregistrée uniquement comme étant présente ou absente, sans mesure standardisée. Cela peut avoir entraîné une mauvaise classification, empêché l’évaluation des relations dose-réponse et probablement rendu plus difficile la détection des associations réelles. Les limites du registre ont restreint l’accès aux données histologiques au-delà de la GS.

**Conclusion:**

Chez les donneurs décédés diabétiques, la présence de protéinurie constitue un marqueur temporel d’un risque accru de perte du greffon à long terme; un élément qui complète les données de la biopsie et les données cliniques. L’évaluation normalisée et quantitative de la protéinurie peut améliorer la stratification du risque et la prise en charge post-transplantation, tout en favorisant une utilisation judicieuse des reins provenant de donneurs diabétiques.

**Lay Summary:**

In kidney transplantation, doctors often worry about using kidneys from people who had diabetes because diabetes can damage the kidneys over time. In this study, we looked at whether protein in the donor’s urine—a sign of kidney damage—could help predict how well the kidney would work after transplant. We studied over 9000 kidney transplant patients in the United States who received kidneys from donors with diabetes. We found that if the donor had protein in their urine, the transplanted kidney was more likely to fail, but not right away. The problems usually happened more than 2.5 years after the transplant. Protein in the urine did not lead to more early problems, like needing dialysis right after surgery. These results suggest that checking for protein in the donor’s urine could help doctors better understand long-term risks and plan follow-up care to improve outcomes after transplant.

## Introduction

The rising prevalence of diabetes mellitus (DM) in the general population is mirrored in potential deceased organ donors.^1^ A history of DM was reported in up to eight percent of deceased donors.^2-5^ Diabetic nephropathy (DN), a leading cause of kidney failure, occurs in 10-30% of patients with DM and is frequently underdiagnosed, even in advanced stages, due to clinical-histologic discordance.^6,7^ Few small cohort studies and case series of deceased donor kidney biopsies report variable prevalence (up to 25%) and severity of DN among kidneys ultimately transplanted, often poorly correlated with glycemic control or DM duration.^5,8-11^ Non-glomerular chronic injury is also frequently observed and linked to worse allograft function and survival.^8^ The implications of donor DN are further complicated by interactions with recipient factors and the post-transplant course influencing the evolution of these lesions, with limited studies documenting histologic trajectories over follow-up.^8-15^

Kidneys from diabetic donors are inconsistently associated with worse early and late outcomes after transplantation, including higher serum creatinine and risks of delayed graft function (DGF), death-censored graft failure (DCGF), all-cause graft failure (ACGF), and mortality.^3,4^ Diabetes is weighted heavily as a risk factor for DCGF in the Kidney Donor Risk Index (KDRI).^16^ Adverse outcomes appear to be especially pronounced with higher DN class, longer DM duration, and extended criteria donor (ECD) status.^2,3,8,17^ Although recipients with diabetes face higher risks of graft failure and mortality, there still appears to be a greater survival benefit receiving a diabetic donor kidney when compared to remaining on the waitlist, particularly for older recipients with longer anticipated wait times.^4,10,18^ Despite potential survival benefits for recipients, kidneys from diabetic donors have twofold higher odds of being discarded.^19^ In 2022, as many as 52% of procured diabetic donor kidneys were ultimately discarded (vs. 21% for non-diabetic donors), with organ or donor quality concerns accounting for over 92% of declined kidney offers in the United States.^20,21^

Recognizing these challenges, efforts to optimize the use of diabetic donor kidneys have emphasized improved risk stratification through pre-implantation biopsies and the prudent selection of recipients most likely to yield the greatest benefit.^22^ Current US guidelines recommend biopsies for donors with DM, yet practices vary widely in biopsy technique, interpretation, and reporting, leading to inconsistent association between biopsy scores and clinical outcomes.^23,24^ Considering these shortcomings of biopsies, clinical markers of kidney function in diabetic donors may offer additional insights into assessment of donor kidney quality. When present, elevated serum creatinine and proteinuria are associated with accelerated decline in kidney function.^25-27^ However, no composite scores have integrated donor DM status alongside biopsy findings and clinical characteristics. There is the need for a more nuanced approach to diabetic donor kidney evaluation which may permit increased utilization of these kidneys while optimizing post-transplant outcomes. We sought to explore the association of proteinuria status among donors with DM who had procurement biopsies, on post-transplant outcomes of graft and patient survival in a large US registry cohort of kidney-alone transplant recipients from 2013 to 2023.

## Methods

### Study Design and Population

This cohort study consisted of all consecutive deceased donor kidney-alone transplant recipients performed in the United States from 28^th^ February 2013 to 2023 where there was a donor history of diabetes mellitus (DM). This study used data from the Scientific Registry of Transplant Recipients (SRTR). This study used data from the Scientific Registry of Transplant Recipients (SRTR). The SRTR data system includes data on all donor, wait-listed candidates, and transplant recipients in the US, submitted by the members of the Organ Procurement and Transplantation Network (OPTN). The Health Resources and Services Administration (HRSA), U.S. Department of Health and Human Services provides oversight to the activities of the OPTN and SRTR contractors. Data is mandatorily reported to SRTR by transplant centers at time of donation and transplantation, and with post-transplant follow-up records at six months, one year, and then annually, until the recipient is re-transplanted, dies, or is lost to follow-up.

Donor history of DM was captured at the time of donation and did not distinguish between type 1 or type 2 DM. Recipients were excluded if there was no available kidney biopsy report done from time of kidney procurement until implantation, and if there was no donor proteinuria testing result available.

Research Ethics Board approval was obtained from the University Health Network and this study was approved by the SRTR.

### Outcomes

The primary outcome of interest was time to death-censored graft failure (DCGF), with patients censored at last follow-up or death with a functioning graft. Secondary outcomes were: (i) time to all-cause graft failure (ACGF), (ii) time to death with graft function (DWGF), and (iii) delayed graft function (DGF). DGF was defined as the need for dialysis within the first week after transplantation.

### Donor Proteinuria Exposure

Donor proteinuria was classified as a binary exposure, present or not present, as captured in the SRTR dataset and reported by transplant centers. Proteinuria measurement methods were not available.

### Statistical Analysis

Analyses were conducted using R (Version 2023). Categorical variables were presented as frequencies and percentages, while continuous variables were presented as means with standard deviations (SD). The distribution of baseline demographics, grouped by proteinuria status, were compared using standardized mean differences (SMD), with SMD > 0.1 suggesting a notable difference between groups.

We utilized the Kaplan-Meier product limit method and log-rank statistic to graphically assess the time-to-event outcomes of DCGF, ACGF, and DWGF by donor proteinuria status.

**Figure 1.**
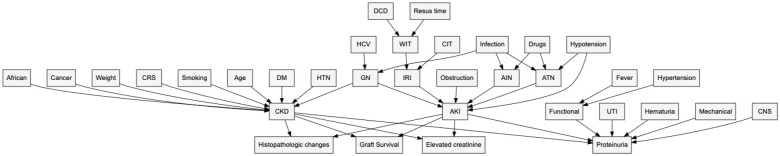
Directed acyclic graph (DAG) showing causal relationships of exposure, outcomes, and covariables. *Note.* AIN = acute interstitial nephritis; AKI = acute kidney injury; ATN = acute tubular necrosis; CIT = cold ischemic time; CKD = chronic kidney disease; CNS = central nervous system pathology; CRS = cardiorenal syndrome; DCD = donation after cardiac death; DM = diabetes mellitus; GN = glomerulonephritis; HCV = hepatitis C virus; HTN = hypertension; IRI = ischemia reperfusion injury; Resus time = resuscitation time; UTI = urinary tract infection; WIT = warm ischemic time.

Multivariable Cox proportional hazards models were used to assess the association between donor proteinuria status and DCGF while adjusting for potential confounders. Covariables included in the Cox models were selected a priori and their relationships were visualized in a directed acyclic graph (DAG). [Figure 1] Candidate covariables included the following donor characteristics: age, biologic sex, height, weight, history of hypertension, donation after cardiac death (DCD) status, stroke as cause of death, and peak serum creatinine prior to donation (in umol/L). If there was <10% missingness, the mechanism was assumed to be at random and multiple imputation with chained equations (MICE) was used (10 imputations); variables with >10% missingness were omitted from models. The proportional hazards assumption was tested with the Grambsch-Therneau test and visually inspected with Schoenfeld residual plots over time. After preliminary analyses, the proportionality assumption was violated in the model for DCGF. All survival analyses were subsequently done in two parts, informed by visual inspection of the survival curve: (i) the “early cohort” analyzed outcomes up to 2.5-years post-transplant, and otherwise censored at 2.5-years, followed by (ii) analysis of a “landmarked cohort” consisting of recipients with functioning allografts landmarked at 2.5-years post-transplant. Hazard ratios (HR) were presented with their associated 95% confidence intervals.

A multivariable logistic regression model was used to assess donor proteinuria as a predictor of DGF and covariables selected using the DAG. Logistic regression models were tested with the Hosmer-Lemeshow test for goodness of fit, variance inflation factor (VIF) for multicollinearity, and Cook’s D for influential observations. Odds ratios (OR) and their associated 95% confidence intervals were presented.

A two-sided *P*-value < .05 was considered statistically significant.

#### Sensitivity analyses

Survival analyses were repeated in recipients whose donor’s insulin-dependence status was documented at transplant, with models adjusted for insulin dependence. Assuming adequate DM treatment in donors, insulin dependence was used as a proxy for DM severity.

To account for recipient factors influencing the decision to accept a donor kidney with a history of DM, models were adjusted for recipient factors including age, biologic sex, wait time on dialysis pre-transplant, any history of diabetes mellitus, history of hypertension, prior solid organ transplant, cold ischemic time, and number of Human Leukocyte Antigen (HLA) mismatches.

#### Subgroup analyses

Two-way interaction between exposure and selected covariables (biologic sex, DCD status, and recipient diabetes status) were tested and excluded from multivariable models if *P*-value < .05.

We also conducted an exploratory analysis for effect measure modification by amount of glomerulosclerosis (GS) on donor kidney biopsy at time of transplant, on the effect of donor proteinuria and DCGF. Univariable Cox proportional hazards models for DCGF were conducted in dichotomized subgroups of recipients based on thresholds of 5%, 10%, and 20% GS among glomeruli sampled. The ratio of hazard ratios (RHR) was reported. Assessment of other histologic lesions (vascular, interstitial fibrosis and tubular atrophy (IFTA)) were precluded by high missingness in the dataset.

## Results

### Study Population

**Figure 2.**
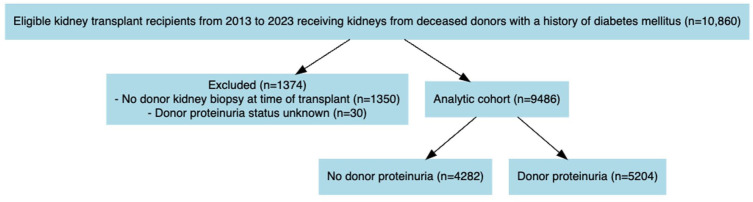
Patient flow in study.

The cohort included 9486 kidney transplant recipients with 33 492 person-years of follow-up (median 3.0 years; IQR 1.0 to 5.0), including 5323 recipients in the 2.5-year landmarked cohort. [Figure 2] Mean recipient age was 59.3 ± 10.9 years and 63% were male.

Donor proteinuria at time of transplant was present in 5204 (54.9%) of donors. Baseline demographics of the analyzed population grouped by donor proteinuria status are shown in Table 1. Donors with proteinuria were more likely to have higher peak serum creatinine (1.47 vs 1.20 umol/L), higher weight, and DCD status. There was no difference in donor insulin dependence (38%) or history of hypertension (70%), and there were no donors with diabetes duration known to exceed 10 years prior to death. Recipients were also balanced in age, sex, and diabetes status (~45%).

**Table 1.** Baseline Demographics of the Cohort by Donor Proteinuria Status and Standardized Mean Differences (SMD) (n = 9486).

	No proteinuria	Proteinuria	Standardized mean difference
**Donor baseline characteristics**			
Age at donation (years)	49.2 (11.2)	48.9 (10.8)	0.034
Male sex (%)	2376 (55.5%)	3103 (59.6%)	0.084
African race (%)	659 (15.4%)	906 (17.4%)	0.055
Caucasian race (%)	3447 (80.5%)	4085 (78.5%)	0.050
Weight (kg)	93.2 (24.7)	98.1 (27.4)	0.188
Height (cm)	170.2 (10.7)	170.7 (10.4)	0.046
Insulin dependence (%) (n = 8551)	1479 (38.4%)	1808 (38.5%)	0.001
History of hypertension (%) (n = 9435)	2992 (70.3%)	3641 (70.3%)	0.001
History of smoking >20 pack-years (%) (n = 9302)	1207 (28.8%)	1493 (29.2%)	0.011
History of myocardial infarction (%) (n = 9367)	376 (8.9%)	481 (9.4%)	0.016
History of cancer (n = 9421)	171 (4.0%)	165 (3.2%)	0.044
History of hepatitis C virus (%) (n = 9480)	193 (4.5%)	198 (3.8%)	0.035
**Donor peri-donation characteristics**			
Donation after cardiac death (%)	900 (21.0%)	1506 (28.9%)	0.184
Stroke as cause of death (%)	1586 (37.0%)	1646 (31.6%)	0.114
Peak serum creatinine (umol/L) (n = 9484)	1.20 (0.87)	1.47 (1.20)	0.258
Urinary tract infection at donation (%)	925 (21.6%)	1176 (22.6%)	0.024
Inotropic support (%) (n = 9477)	1840 (43.0%)	2032 (39.0%)	0.080
**Recipient characteristics**			
Age at transplant (years)	59.0 (11.0)	59.5 (10.8)	0.045
Male sex (%)	2667 (62.3%)	3324 (63.9%)	0.033
Wait time pre-transplant (years)	2.51 (2.28)	2.36 (2.28)	0.066
History of diabetes mellitus (%) (n = 9355)	1871 (44.4%)	2393 (46.6%)	0.044
Type of diabetes mellitus (%) (n = 4264)			0.045
- Type 1	109 (2.6%)	133 (2.6%)	
- Type 2	1762 (41.8%)	2260 (44.0%)	

*Note.* Mean (standard deviation) is presented for continuous values. Frequency (percentage) is presented for categorical values.

### Death-Censored Graft Failure

There were 1315 DCGF events observed during the follow-up period, with 534 of these events occurring beyond 2.5 years post-transplant.

**Figure 3.**
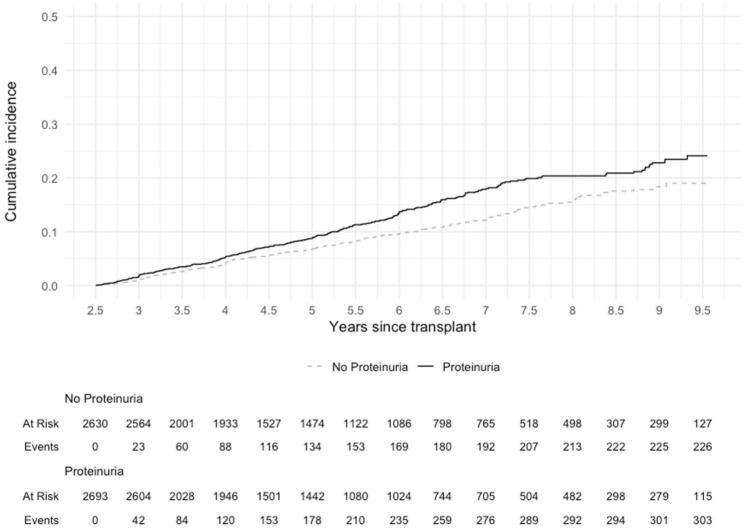
Cumulative incidence of death-censored graft failure in landmarked cohort of kidney transplant patients by donor proteinuria status (log rank *P* < .001). *Note.* The x-axis was truncated to start at 913 days as the landmarked cohort had follow-up starting at 913 days post-transplant.

**Figure 4.**
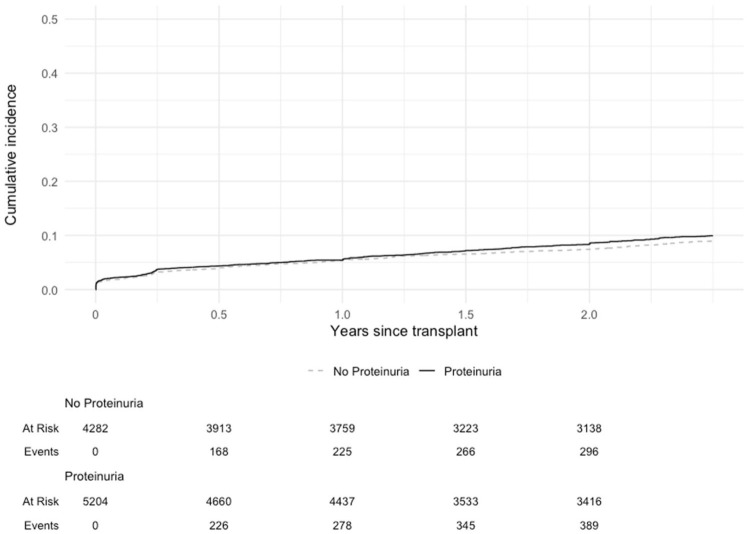
Cumulative incidence of death-censored graft failure in the early cohort of kidney transplant patients by donor proteinuria status (log rank *P* = .10).

Donor proteinuria was associated with an increased risk of DCGF events in the landmarked cohort using an unadjusted Kaplan-Meier analysis (*P* < .001) and in the multivariable Cox proportional hazards model (HR 1.36 [95% CI: 1.15, 1.62]) after adjusting for clinically relevant donor characteristics (Figure 3; Supplemental Table 1; Table 2). Donor proteinuria was not clearly associated with an increased risk of DCGF in the early cohort, both in unadjusted and adjusted models (HR 1.14 [95% CI: 0.99, 1.32]; Figure 4; Supplemental Table 1; Table 2).

**Table 2.** Multivariable Cox Proportional Hazards Models for Death-Censored Graft Failure, All-Cause Graft Failure, and Death With Graft Function, in the Early (n = 9486) and Landmarked (n = 5323) Patient Cohorts.

Cohort	DCGF HR (95%) CI	ACGF HR (95% CI)	DWGF HR (95% CI)
Early cohort	1.14 (0.99, 1.32)	1.10 (0.99, 1.22)	1.06 (0.92, 1.22)
Landmarked cohort	1.35 (1.14, 1.61)	1.18 (1.06, 1.31)	1.08 (0.95, 1.24)

*Note.* DCGF events: 781 in early cohort, 534 in landmarked cohort. ACGF events: 1608 in early cohort, 1403 in landmarked cohort. DWGF events: 827 in early cohort, 869 in landmarked cohort. The models were adjusted for donor age, sex, weight, height, history of hypertension, and maximum serum creatinine.

### All-Cause Graft Failure

There were 3011 ACGF events including 1403 events in the 2.5-year landmarked cohort. Donor proteinuria was associated with an increased risk of ACGF beyond 2.5 years post-transplant (HR 1.18 [95% CI: 1.06, 1.31]), but not in the early cohort (HR 1.10 [95% CI: 0.99, 1.22]; Supplemental Table 1; Table 2).

### Death With Graft Function

There were 1696 patients who died with graft function, with 869 deaths occurring more than 2.5 years post-transplant. Donor proteinuria was not associated with DWGF in the early (HR 1.06 [95% CI: 0.92, 1.22]) or landmarked (HR 1.08 [95% CI: 0.95, 1.24]) cohorts (Supplemental Table 1; Table 2).

### Delayed Graft Function

DGF occurred in 33.2% (3339 of 9484) recipients and was not associated with donor proteinuria status (OR 1.08 [95% CI: 0.99, 1.18]).

### Sensitivity Analyses

Survival analyses were repeated in the sub-cohort of 8551 recipients where donor history of insulin dependence was available at time of transplant; insulin dependence was reported in 38% of donors. The effect of donor proteinuria was similar to the primary analyses in a model adjusted for donor insulin dependence, and a model adjusted for recipient and donor-recipient factors (Supplemental Table 2). Insulin dependence was also independently associated with an increased risk of DCGF in both the early (HR 1.35 [95% CI: 1.16, 1.57]) and landmarked (HR 1.32 [95% CI: 1.10, 1.59]) cohorts, and ACGF in early (HR 1.19 [95% CI: 1.07, 1.33]) and landmarked (HR 1.23 [95% CI: 1.10, 1.38]) cohorts.

Primary analyses were unchanged by the addition of recipient characteristics as covariables in multivariable models (Supplemental Table 2).

### Subgroup Analyses

There was no statistically significant interaction between donor proteinuria and biological sex, DCD status, or recipient diabetes status when recipient characteristics were included in the multivariable Cox models.

The effect of donor proteinuria on DCGF beyond 2.5 years post-transplant was more pronounced in recipients of donor kidneys with GS below versus above the threshold, and this was observed at all GS thresholds tested: RHR 1.35 at 5% GS, 1.45 at 10% GS, and 1.82 at 20% GS (Supplemental Table 3).

## Discussion

In this large cohort of kidney transplant recipients from diabetic donors, we found that donor proteinuria was associated with worse death-censored and all-cause graft outcomes in the later post-transplant period beyond 2.5 years. The absence of significant associations with DGF or early graft failure suggests a time-dependent effect of proteinuria, potentially reflecting chronic injury pathways that progress over time. These findings were robust to adjustment for donor insulin dependence—a proxy for diabetes severity—and recipient characteristics, with no difference by recipient diabetes status.

The presence of proteinuria in diabetic kidney donors was associated with worse graft survival in the later post-transplant period. Although prior studies have largely overlooked the impact of donor proteinuria on post-transplant outcomes, a recent Swiss registry study of 1725 all-comer deceased donors reported proteinuria (urine protein-to-creatinine ratio >15 mg/mmol) in three quarters of donor candidates, but no association with 1-year mortality or graft function and survival.^28,29^ Our cohort, which was enriched for diabetic donors more likely to have clinically significant proteinuria, found that proteinuria was a marker of late graft failure even after adjusting for clinically relevant donor characteristics.

The delayed effect on graft survival may reflect the progression of early donor DN lesions. All donor kidneys had a pre-implantation biopsy, and severe histologic lesions linked to early graft failure would have likely precluded transplantation, selecting for kidneys with less advanced lesions that may evolve over time. While case reports documented resolution of donor DN lesions in non-diabetic recipients,^12-14^ other small observational studies found stability or progression within one year post-transplant irrespective of recipient diabetes status or glycemic exposure.^8,10,11^ However, these studies were limited by short-term follow-up and lack of correlation with clinical outcomes. Although we found no interaction with recipient diabetes status, used as a surrogate for glycemic exposure post-transplant, we could not account for glycemic control, post-transplant diabetes, or diabetes-related comorbidities. This underscores the need for studies evaluating the long-term evolution of donor DN lesions. Taken together, these findings suggest that proteinuria may serve as a surrogate for donor-derived kidney disease not captured by current biopsy practices, which progresses slowly but contributes significantly to late graft failure.

All donor kidneys in this study underwent pre-implantation biopsy, yet the association of proteinuria with late graft failure highlights limitations in biopsy utility for predicting long-term outcomes. Autopsy studies suggest that proteinuria is more strongly associated with kidney function and IFTA than with classic DN lesions.^6^ Donor kidneys with severe glomerular or interstitial DN lesions, more closely linked to serum creatinine, may have been excluded from transplantation based on thresholds for creatinine or GS. Although GS >20% has been inconsistently linked to worse graft survival, GS remains a key factor in kidney discard.^30-33^ Notably, we observed that the association between proteinuria and late graft failure was stronger in kidneys with lower levels of GS. Proteinuria in this cohort may therefore reflect alternative pathways like early DN or chronic IFTA, which could be missed on pre-implantation biopsies due to sampling error and variability across observers or biopsy techniques, or may be overlooked in clinical decisions due to scarce guidelines and poor prognostic performance of histologic scoring systems.^24,34-37^ This would be consistent with our findings as early or scarring-related injuries could progress over time to cause late graft failure, and IFTA would not be modified by recipient diabetes status. While current OPTN guidelines recommend standardized biopsy reporting, including percent GS and IFTA, only GS is reliably captured in the SRTR registry and was available for analyses.^38^ These inconsistencies emphasize the need for standardized biopsy protocols and comprehensive reporting frameworks that incorporate DN-specific features, such as diabetic glomerulopathy and IFTA, to enhance donor kidney evaluation and selection.

Our findings suggest that proteinuria is a helpful marker of diabetic donor kidney quality, complementing traditional metrics such as donor demographics and peak serum creatinine, even in a population where pre-implantation biopsies were performed. The observed association between proteinuria and late graft failure supports its routine assessment, but given that all kidneys in this study were ultimately transplanted, applying these findings without nuance could inadvertently increase organ discard. Whether proteinuria could help guide decisions about kidneys that were previously declined remains unknown. Instead, donor proteinuria assessment may offer an opportunity to tailor post-transplant care in high-risk recipients—for example, through more intensive monitoring, risk factor management, or earlier initiation of renin-angiotensin-aldosterone-system inhibitors. Current OPTN guidelines recommend biopsies for all diabetic donors, and proteinuria measurement could similarly be standardized.^23^ However, this study alone cannot guide decisions regarding the best method for measuring proteinuria. Proteinuria was reported at the discretion of centers, with minimal missing data (Figure 1), but the specific measurement methods and thresholds used to define its presence were unknown. This binary classification of proteinuria limited our ability to evaluate dose-response effects, potentially underestimating the association with outcomes by including lower, less clinically relevant levels of proteinuria. While any proteinuria was significantly associated with worse outcomes, quantitative methods such as urine albumin-to-creatinine ratio (UACR) are preferred over qualitative approaches like urinalysis, aligning with best practices in nephrology. UACR is more sensitive for detecting clinically relevant proteinuria in DN and other kidney diseases, reflecting its utility in chronic kidney disease care.^27^ However, dichotomized proteinuria status may allow generalizability of these findings to resource-limited jurisdictions where proteinuria quantification is unavailable. Future studies should explore the optimal number, timing, and thresholds for proteinuria tests, as well as the integration of standardized measurements into diabetic donor kidney evaluations. Addressing these gaps could enhance donor evaluation and improve long-term outcomes without compromising organ utilization.

This study provides valuable insights into the underexplored and underutilized population of diabetic donor kidneys, leveraging a large, contemporary national registry cohort spanning 10 years. Nearly all diabetic donors underwent pre-implantation biopsy, consistent with current practices and OPTN guidelines recommending biopsies for donors with diabetes or a single elevated HbA1c (≥6.5%).^23^ A survey of 95 US transplant centers further highlighted that 51% consider donor diabetes, especially durations of 5–10 years, a criterion for biopsy.^35^ By focusing on this cohort, our study addresses a gap in transplant research and provides a foundation for understanding long-term outcomes of diabetic donor kidneys. The findings were adjusted for key donor characteristics, including insulin dependence as a proxy for diabetes severity (including type 1 diabetes or insulin-dependent type 2 diabetes), and recipient factors, offering a comprehensive assessment of proteinuria’s role in post-transplant outcomes. Subgroup analyses accounted for recipient diabetes status as a surrogate for ongoing glycemic exposure, though we could not capture the post-transplant course. These strengths enhance the generalizability of our findings and underscore the importance of evaluating diabetic donor kidney function.

However, as an observational study, our findings are subject to residual and unmeasured confounding. Selection bias may also be present, as only kidneys deemed suitable for transplantation were included, likely excluding diabetic donor kidneys with more severe dysfunction or significant histologic findings identified on pre-implantation biopsies. The exclusion of kidneys expected to confer poor post-transplant outcomes therefore leaves a cohort of eligible diabetic donor kidneys where there is uncertainty about how best they may be utilized, which is a key population of interest. Additionally, the use of registry data introduced limitations in the consistency and granularity of reported outcomes.^39-41^ Donor proteinuria exposure was susceptible to measurement error, including in the presence of acute kidney injury, potentially underestimating its true impact by including lower levels of proteinuria with less clinical relevance. False-positive proteinuria, from factors such as hematuria, urinary tract infections, or foley catheters in deceased donors, may have also biased the results toward the null, though this ultimately strengthens conclusions that donor proteinuria captured here was a negative prognostic marker when present.^42,43^ Nonetheless, the diabetic donor cohort was enriched for true proteinuria related to kidney disease, supporting the clinical relevance of these findings. Future studies with standardized and quantified proteinuria measurement with detailed histologic data are essential to better assess diabetic donor kidneys and improve graft survival outcomes.

These findings emphasize the need for a more nuanced evaluation of diabetic donor kidneys and suggest that proteinuria, while not an immediate risk factor for graft loss, may herald a risk for cumulative injury that manifests in the long-term post-transplant period. Incorporating standardized and quantitative proteinuria measurements into routine donor assessments, alongside comprehensive histologic evaluation and clinical data, could improve risk stratification and enhance the utility of diabetic donor kidneys. Future studies should explore how integrating these markers into clinical decision-making might optimize outcomes while reducing kidney discard, ultimately improving access to transplantation for patients on the waitlist.

## Supplemental Material

sj-docx-1-cjk-10.1177_20543581261424568 – Supplemental material for Proteinuria in Deceased Diabetic Donors and Kidney Transplant Outcomes Supplemental material, sj-docx-1-cjk-10.1177_20543581261424568 for Proteinuria in Deceased Diabetic Donors and Kidney Transplant Outcomes by Christie Rampersad and S. Joseph Kim in Canadian Journal of Kidney Health and Disease

## References

[1] CDC. Prevention USA 2024. https://www.cdc.gov/diabetes/php/data-research/index.html. Accessed November 10, 2024.

[2] Mohan S Tanriover B Ali N , et al. Availability, utilization and outcomes of deceased diabetic donor kidneys; analysis based on the UNOS registry. Am J Transplant. 2012;12(8):2098-2105. 22758926 10.1111/j.1600-6143.2012.04167.x PMC3409306

[3] Ahmad M Cole EH Cardella CJ , et al. Impact of deceased donor diabetes mellitus on kidney transplant outcomes: a propensity score-matched study. Transplantation. 2009;88(2):251-260. 19623022 10.1097/TP.0b013e3181ac68a9

[4] Cohen JB Bloom RD Reese PP Porrett PM Forde KA Sawinski DL. National outcomes of kidney transplantation from deceased diabetic donors [published online ahead of print October 21, 2015]. Kidney Int. 2015. doi:10.1038/ki.2015.325. PMC4840104 26489026

[5] Truong LD Suki WN Gaber LW Gaber OA Khan F. Kidney donors with diabetes: renal biopsy findings at time of transplantation and their significance. Transplant Direct. 2019;5(7):e465. 10.1097/TXD.0000000000000903 PMC6616142 31334339

[6] Klessens CQ Woutman TD Veraar KA , et al. An autopsy study suggests that diabetic nephropathy is underdiagnosed. Kidney Int. 2016;90(1):149-156. 27165826 10.1016/j.kint.2016.01.023

[7] Krolewski AS Skupien J Rossing P Warram JH. Fast renal decline to end-stage renal disease: an unrecognized feature of nephropathy in diabetes. Kidney Int. 2017;91(6):1300-1311. 28366227 10.1016/j.kint.2016.10.046 PMC5429989

[8] Gilbert A Scott D Stack M , et al. Long-standing donor diabetes and pathologic findings are associated with shorter allograft survival in recipients of kidney transplants from diabetic donors. Mod Pathol. 2022;35(1):128-134. 34584213 10.1038/s41379-021-00927-2

[9] Hsu CT Wen MC Chiu HF , et al. Ongoing donor-transmitted diabetic kidney disease in kidney transplant recipients with fair sugar control: a single center retrospective study. BMC Nephrol. 2020;21(1):458. 33143634 10.1186/s12882-020-02132-w PMC7640448

[10] Khan FN Truong LD Nguyen DT , et al. Outcomes of kidney transplantation using deceased donors with history of diabetes. Clin Transplant. 2020;34(2):e13775. 10.1111/ctr.13775 31863607

[11] Lee KW Sim J Park SSW , et al. Recoverability of diabetic nephropathy of donor kidney after kidney transplantation. Transpl Int. 2022;35:10714. 36187463 10.3389/ti.2022.10714 PMC9519853

[12] Abouna GM Al-Adnani MS Kremer GD Kumar SA Daddah SK Kusma G. Reversal of diabetic nephropathy in human cadaveric kidneys after transplantation into non-diabetic recipients. Lancet. 1983;2(8362):1274-1276. 6139620 10.1016/s0140-6736(83)91151-0

[13] Okubo N Miki K Yamanouchi M , et al. Kidney transplantation from a diabetic donor to a nondiabetic recipient: a case report. Transplant Proc. 2022;54(10):2748-2753. 36424226 10.1016/j.transproceed.2022.09.024

[14] Harada S Ushigome H Nishimura A , et al. Histological reversibility of diabetic nephropathy after kidney transplantation from diabetic donor to non-diabetic recipient. Nephrology. 2015;20 suppl 2:40-44. 10.1111/nep.12451 26031585

[15] Visona I Franco MF. Deceased donor with diabetic nephropathy: case report of four kidney recipients. Austin J Nephrol Hypertens. 2017;4(1):1064.

[16] Rao PS Schaubel DE Guidinger MK , et al. A comprehensive risk quantification score for deceased donor kidneys: the kidney donor risk index. Transplantation. 2009;88(2):231-236. 19623019 10.1097/TP.0b013e3181ac620b

[17] Chen Q Guo J Han S , et al. The impact of donor diabetes on recipient postoperative complications, renal function, and survival rate in deceased donor kidney transplantation: a single-center analysis. Ren Fail. 2024;46(2):2391067. 10.1080/0886022X.2024.2391067 PMC11346333 39177237

[18] Cohen JB Eddinger KC Locke JE Forde KA Reese PP Sawinski DL. Survival benefit of transplantation with a deceased diabetic donor kidney compared with remaining on the waitlist. Clin J Am Soc Nephrol. 2017;12(6):974-982. 28546439 10.2215/CJN.10280916 PMC5460711

[19] Mohan S Chiles MC Patzer RE , et al. Factors leading to the discard of deceased donor kidneys in the United States. Kidney Int. 2018;94(1):187-198. 29735310 10.1016/j.kint.2018.02.016 PMC6015528

[20] Lentine KL Smith JM Lyden GR , et al. OPTN/SRTR 2022 annual data report: kidney. Am J Transplant. 2024;24(2S1):S19-S118. 10.1016/j.ajt.2024.01.012 38431360

[21] Husain SA King KL Pastan S , et al. Association between declined offers of deceased donor kidney allograft and outcomes in kidney transplant candidates. JAMA Netw Open. 2019;2(8):e1910312. 10.1001/jamanetworkopen.2019.10312 PMC6724162 31469394

[22] Thongprayoon C Miao J Jadlowiec CC , et al. Differences between kidney transplant recipients from deceased donors with diabetes mellitus as identified by machine learning consensus clustering. J Pers Med. 2023;13(7):1094. 37511707 10.3390/jpm13071094 PMC10381319

[23] Committee OKT 2022. https://optn.transplant.hrsa.gov/policies-bylaws/public-comment/establish-minimum-kidney-donor-criteria-to-require-biopsy/. Accessed November 10, 2024.

[24] Wang CJ Wetmore JB Crary GS Kasiske BL. The donor kidney biopsy and its implications in predicting graft outcomes: a systematic review. Am J Transplant. 2015;15(7):1903-1914. 25772854 10.1111/ajt.13213

[25] Gerstein HC Mann JF Yi Q , et al. Albuminuria and risk of cardiovascular events, death, and heart failure in diabetic and nondiabetic individuals. JAMA. 2001;286(4):421-426. 11466120 10.1001/jama.286.4.421

[26] Tangri N Stevens LA Griffith J , et al. A predictive model for progression of chronic kidney disease to kidney failure. JAMA. 2011;305(15):1553-1559. 21482743 10.1001/jama.2011.451

[27] Kidney Disease: Improving Global Outcomes CKDWG. KDIGO 2024 clinical practice guideline for the evaluation and management of chronic kidney disease. Kidney Int. 2024;105(4S):S117-S314. 10.1016/j.kint.2023.10.018 38490803

[28] Kuhn C Born A Karolin A , et al. Relevance of deceased donor proteinuria for kidney transplantation: a comprehensive national cohort study. Clin Transplant. 2022;36(4):e14574. 10.1111/ctr.14574 35124857

[29] Schaapherder AF Kaisar M Mumford L , et al. Donor characteristics and their impact on kidney transplantation outcomes: results from two nationwide instrumental variable analyses based on outcomes of donor kidney pairs accepted for transplantation. eClinicalMedicine. 2022;50:101516. 35784435 10.1016/j.eclinm.2022.101516 PMC9240982

[30] Gaber LW Moore LW Alloway RR Amiri MH Vera SR Gaber AO. Glomerulosclerosis as a determinant of posttransplant function of older donor renal allografts. Transplantation. 1995;60(4):334-339. 7652761 10.1097/00007890-199508270-00006

[31] Edwards EB Posner MP Maluf DG Kauffman HM. Reasons for non-use of recovered kidneys: the effect of donor glomerulosclerosis and creatinine clearance on graft survival. Transplantation. 2004;77(9):1411-1415. 15167600 10.1097/01.tp.0000123080.19145.59

[32] Sung RS Christensen LL Leichtman AB , et al. Determinants of discard of expanded criteria donor kidneys: impact of biopsy and machine perfusion. Am J Transplant. 2008;8(4):783-792. 18294347 10.1111/j.1600-6143.2008.02157.x

[33] Wang CJ Wetmore JB Wey A Miller J Snyder JJ Israni AK. Impact of donor kidney biopsy on kidney yield and posttransplant outcomes. Am J Transplant. 2023;23(3):387-392. 36695677 10.1016/j.ajt.2022.11.020

[34] Liapis H Gaut JP Klein C , et al. Banff histopathological consensus criteria for preimplantation kidney biopsies. Am J Transplant. 2017;17(1):140-150. 27333454 10.1111/ajt.13929 PMC6139430

[35] Lentine KL Naik AS Schnitzler MA , et al. Variation in use of procurement biopsies and its implications for discard of deceased donor kidneys recovered for transplantation. Am J Transplant. 2019;19(8):2241-2251. 30809941 10.1111/ajt.15325

[36] Emmons BR Husain SA King KL Adler JT Mohan S . Variations in deceased donor kidney procurement biopsy practice patterns: a survey of U.S. Clin Transplant. 2021;35(9):e14411. 10.1111/ctr.14411 PMC8556234 34196034

[37] Jadav P Mohan S Husain SA. Role of deceased donor kidney procurement biopsies in organ allocation. Curr Opin Nephrol Hypertens. 2021;30(6):571-576. 34545039 10.1097/MNH.0000000000000746 PMC8490331

[38] Committee OKT 2022. https://optn.transplant.hrsa.gov/media/sqyirv4c/standardize-kidney-biopsy-reporting-and-data-collection_winter-2022-pc.pdf. Accessed November 10, 2024.

[39] Massie AB Kucirka LM Segev DL. Big data in organ transplantation: registries and administrative claims. Am J Transplant. 2014;14(8):1723-1730. 25040084 10.1111/ajt.12777 PMC4387865

[40] Karam V Gunson B Roggen F , et al. Quality control of the European Liver Transplant Registry: results of audit visits to the contributing centers. Transplantation. 2003;75(12):2167-2173. 12829939 10.1097/01.TP.0000080271.20145.07

[41] Yu M King KL Husain SA , et al. Discrepant outcomes between national kidney transplant data registries in the United States. J Am Soc Nephrol. 2023;34(11):1863-1874. 37535362 10.1681/ASN.0000000000000194 PMC10631598

[42] Parker JL Kirmiz S Noyes SL , et al. Reliability of urinalysis for identification of proteinuria is reduced in the presence of other abnormalities including high specific gravity and hematuria. Urol Oncol. 2020;38(11):853.e9-853.e15. 10.1016/j.urolonc.2020.06.035 32739229

[43] Haider MZ Aslam A. Proteinuria. Treasure Island, FL: StatPearls; 2024.

