# Supplementary material for: Proteinuria in Deceased Diabetic Donors and Kidney Transplant Outcomes
Source: Can J Kidney Health Dis. 2026 Feb 26;13:20543581261424568. doi: 10.1177/20543581261424568 (PMC12949268; doi:10.1177/20543581261424568)
Supplement: sj-docx-1-cjk-10.1177_20543581261424568 – Supplemental material for Proteinuria in Deceased Diabetic Donors and Kidney Transplant Outcomes [file sj-docx-1-cjk-10.1177_20543581261424568.docx]

**Supplemental Figure 1. Schoenfeld residuals from the unadjusted Cox proportional hazards model for death-censored graft failure over full follow-up.**


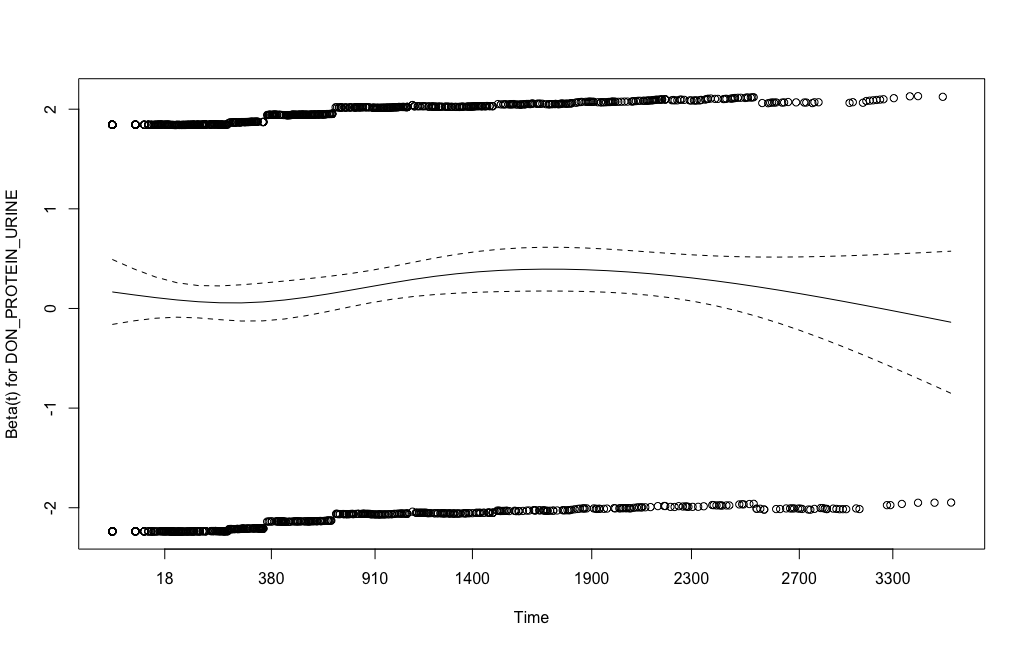


- Horizontal lines represent scaled residual smoothers.

**Supplemental Table 1. Univariable Cox proportional hazards models for death-censored graft failure, all-cause graft failure, and death with graft function, in the early (n=9486) and landmarked (n=5323) patient cohorts.**

| Cohort | DCGF  HR (95%) CI | ACGF  HR (95% CI) | DWGF  HR (95% CI) |
| --- | --- | --- | --- |
| Early cohort | 1.11 (0.97, 1.28) | 1.08 (0.98, 1.20) | 1.06 (0.92, 1.22) |
| Landmarked cohort | 1.35 (1.14, 1.61) | 1.19 (1.07, 1.33) | 1.11 (0.97, 1.26) |
| - DCGF events: 781 in early cohort, 534 in landmarked cohort. - ACGF events: 1608 in early cohort, 1403 in landmarked cohort. - DWGF events: 827 in early cohort, 869 in landmarked cohort. | | | |

**Supplemental Table 2. Sensitivity analyses for survival models for death-censored graft failure, all-cause graft failure, and death with graft function after adjusting for: (i) recipient characteristics; and (ii) donor insulin-dependence status.**

|  | DCGF | | ACGF | | DWGF | |
| --- | --- | --- | --- | --- | --- | --- |
|  | HR (95% CI) | p-value | HR (95% CI) | p-value | HR (95% CI) | p-value |
| Models adjusted for recipient characteristics* | | | | | | |
| Early cohort (n=9486) | 1.15 (1,00, 1.33) | 0.06 | 1.10 (0.99, 1.21) | 0.08 | 1.04 (0.91, 1.20) | 0.55 |
| Landmarked cohort (n=5323) | **1.35 (1.14, 1.61)** | **<0.001** | **1.18 (1.06, 1.31)** | **0.003** | 1.07 (0.93, 1.23) | 0.34 |
| Models adjusted for donor insulin-dependence status^+^ | | | | | | |
| Early cohort (n=8551) | 1.12 (0.97, 1.31) | 0.12 | 1.10 (0.99, 1.22) | 0.07 | 1.08 (0.93, 1.25) | 0.29 |
| Landmarked cohort (n=4878) | **1.32 (1.10, 1.58)** | **0.003** | **1.18 (1.06, 1.32)** | **0.003** | 1.11 (0.96, 1.27) | 0.16 |
| - Bold values denote statistical significance. - Survival models were Cox proportional hazards models. - All models were adjusted for donor age, sex, weight, height, history of hypertension, and maximum serum creatinine. - * Models were adjusted for recipient age at transplant, biologic sex, wait time on dialysis pre-transplant, prior transplant, any history of diabetes mellitus, cold ischemic time, and number of HLA mismatches. - ^+^ Models were adjusted for donor insulin-dependence status. | | | | | | |

**Supplemental Table 3. Effect measure modification of percentage glomerulosclerosis in donor kidney biopsy at time of transplant in subgroups of patients above and below thresholds of 5%, 10%, and 20% glomerulosclerosis in the early and landmarked subcohorts, on effect of donor proteinuria on death-censored graft failure in unadjusted survival models.**

| Threshold of GS percentage | Subcohort | Subgroup | Number of patients, number of DCGF events | Hazard ratio  (95% CI) | Ratio of hazard ratios (RHR) |
| --- | --- | --- | --- | --- | --- |
| 5% | Early cohort | Below threshold | 4860 pts, 311 events | 1.09 (0.87, 1.36) | 0.96 |
|  |  | Above threshold | 4613 pts, 468 events | 1.13 (0.94, 1.36) |  |
|  | Landmarked cohort | Below threshold | 2779 pts, 248 events | **1.58 (1.23, 2.04)** | 1.35 |
|  |  | Above threshold | 2535 pts, 285 events | 1.17 (0.93, 1.48) |  |
| 10% | Early cohort | Below threshold | 7066 pts, 521 events | 1.11 (0.94, 1.33) | 1.01 |
|  |  | Above threshold | 2407 pts, 258 events | 1.10 (0.86, 1.41) |  |
|  | Landmarked cohort | Below threshold | 3988 pts, 378 events | **1.51 (1.23, 1.85)** | 1.45 |
|  |  | Above threshold | 1326 pts, 155 events | 1.04 (0.76, 1.43) |  |
| 20% | Early cohort | Below threshold | 8839 pts, 704 events | 1.11 (0.95, 1.28) | 0.96 |
|  |  | Above threshold | 634 pts, 75 events | 1.16 (0.73, 1.85) |  |
|  | Landmarked cohort | Below threshold | 4976 pts, 491 events | **1.42 (1.18, 1.69)** | **1.82** |
|  |  | Above threshold | 338 pts, 42 events | 0.78 (0.43, 1.44) |  |
| - Bold values denote statistical significance. - There was a significant interaction between donor proteinuria and percentage of glomerulosclerosis >20% in the landmarked cohort, p=0.04. Other interactions were non-significant, p>0.05. - Survival models were unadjusted Cox proportional hazards models. - Ratio of hazard ratios was calculated as the ratio of hazard ratios below versus above the GS threshold. RHR was only calculated if at least one HR in the pair was statistically significant. | | | | | |
